# Development of optical sensor for water in acetonitrile based on propeller-structured BODIPY-type pyridine–boron trifluoride complex†

**DOI:** 10.1039/d0ra06569b

**Published:** 2020-09-14

**Authors:** Shuhei Tsumura, Kazuki Ohira, Keiichi Imato, Yousuke Ooyama

**Affiliations:** a Department of Applied Chemistry, Graduate School of Engineering, Hiroshima University 1-4-1 Kagamiyama Higashi-Hiroshima 739-8527 Japan kimato@hiroshima-u.ac.jp yooyama@hiroshima-u.ac.jp +81-82-424-5494

## Abstract

A propeller-structured 3,5,8-trithienyl-BODIPY-type pyridine–boron trifluoride complex, ST-3-BF_3_, which has three units of 2-(pyridin-4-yl)-3-(thiophen-2-yl)acrylonitrile at the 3-, 5-, and 8-positions on the BODIPY skeleton, was designed and developed as an intramolecular charge transfer (ICT)-type optical sensor for the detection of a trace amount of water in acetonitrile. The characterization of ST-3-BF_3_ was successfully determined by FTIR, ^1^H and ^11^B NMR measurements, high-resolution mass spectrometry (HRMS) analysis, thermogravimetry-differential thermal analysis (TG-DTA), photoabsorption and fluorescence spectral measurements, and density functional theory (DFT) calculations. ST-3-BF_3_ showed a broad photoabsorption band in the range of 600 to 800 nm, which is assigned to the S_0_ → S_1_ transition of the BODIPY skeleton with the expanded π-conjugated system over the 2-(pyridin-4-yl)-3-(thiophen-2-yl)acrylonitrile units at the 3-, 5-, and 8-positions onto the BODIPY core. In addition, a photoabsorption band was also observed in the range of 300 to 550 nm, which can be assigned to the ICT band between the 2-(pyridin-4-yl)-3-(thiophen-2-yl)acrylonitrile units at 3-, 5-, and 8-positions and the BODIPY core. ST-3-BF_3_ exhibited a characteristic fluorescence band originating from the BODIPY skeleton at around 730 nm. It was found that by addition of a trace amount of water to the acetonitrile solution of ST-3-BF_3_, the photoabsorption band at around 415 nm and the fluorescence band at around 730 nm increased linearly as a function of the water content below only 0.2 wt%, which could be ascribed to the change in the ICT characteristics due to the dissociation of ST-3-BF_3_ into ST-3 by water molecules. Thus, this work demonstrated that the 3,5,8-trithienyl-BODIPY-type pyridine–boron trifluoride complex can act as a highly-sensitive optical sensor for the detection of a trace amount of water in acetonitrile.

## Abstract 2

Propeller-structured 3,5,8-trithienyl-BODIPY-type pyridine–boron trifluoride complex, ST-3-BF_3_, has been developed as an intramolecular charge transfer (ICT)-type optical sensor for the detection of a trace amount of water in acetonitrile.

## Graphical abstract

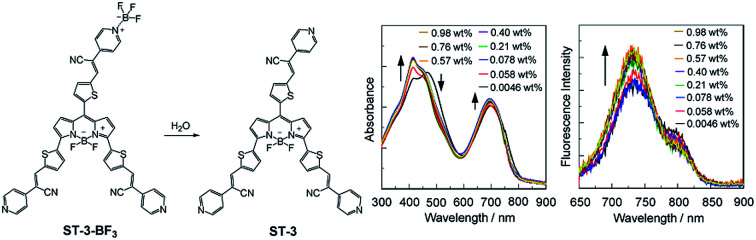

## Introduction

**Fig. 1:**
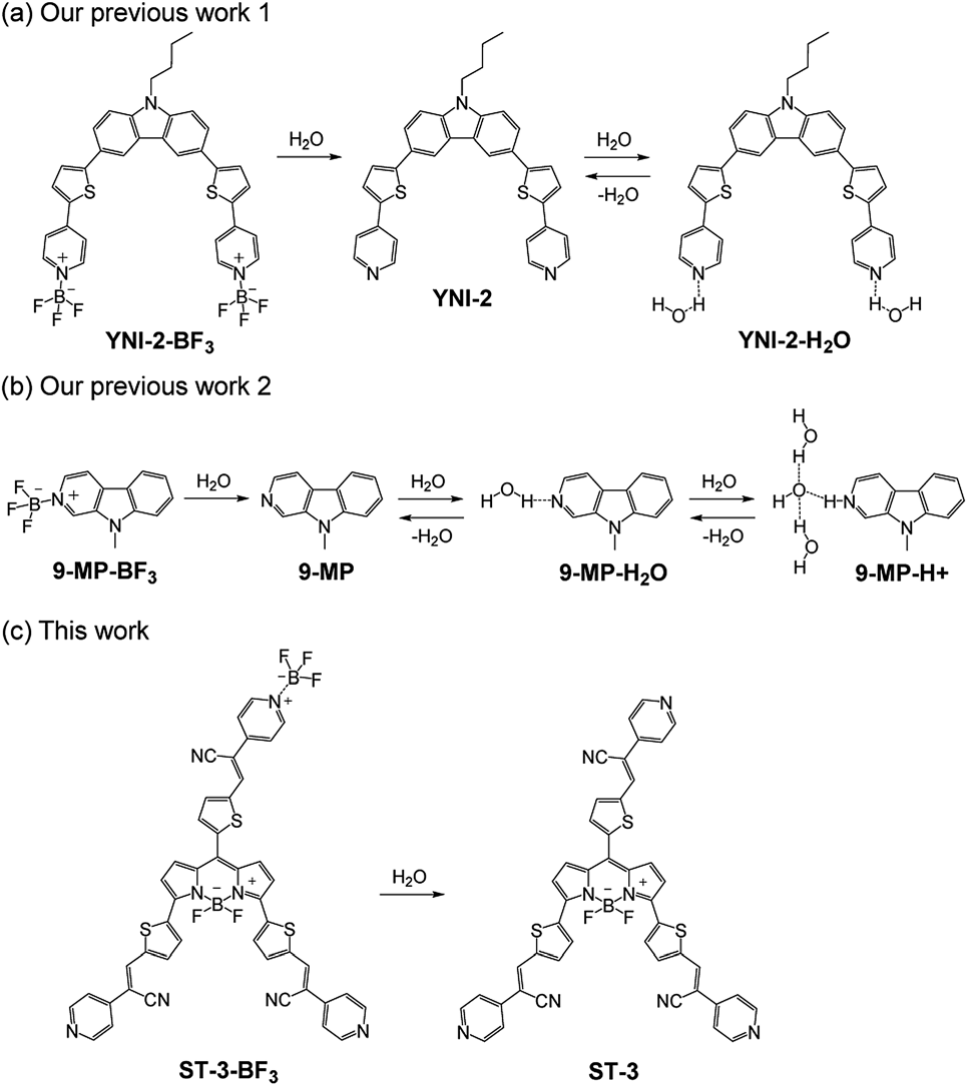
Proposed mechanisms of colorimetric and fluorescent sensors (a) YNI-2-BF_3_, (b) 9-MP-BF_3_, and (c) propeller-structured BODIPY-type pyridine–boron trifluoride complex ST-3-BF_3_ for the detection of water in solvents.

Optical methods utilizing colorimetric and fluorescent sensors for visualization as well as detection and quantification of water in samples and products, such as solutions, solids, and gases or water on substrate surfaces have been of considerable scientific and practical concern in recent years, because of not only fundamental studies in photochemistry, photophysics, and analytical chemistry, but also their potential applications to environmental and quality control monitoring systems and industry.^1–9^ In fact, to date, some kinds of colorimetric and fluorescent sensors for water based on ICT (intramolecular charge transfer),^10,11^ PET (photo-induced electron transfer),^12,13^ or ESIP (excited state intramolecular proton transfer)^14^ have been designed and developed. Among them, the ICT-type sensor, which has a donor–π–acceptor (D–π–A) structure with photoabsorption and fluorescence properties originating from the ICT excitation from the electron-donating (D) moiety to the electron-accepting (A) moiety, allows colorimetric and ratiometric fluorescence measurements, which are preferable because the ratio of photoabsorption or fluorescence intensities at two wavelengths is in fact independent of the total concentration of the sensor, photobleaching, fluctuations in light source intensity, sensitivity of the instrument, *etc.* Indeed, in ICT-type sensors based on a D–π–A structure for detecting cations, anions, and neutral organic species, the dipole moment and electronic structure changed due to the intermolecular interaction (electrostatic interaction) between the electron-donating or electron-accepting moiety of the sensors and the species, resulting in changes in photoabsorption, fluorescence (intensity and wavelength), and electrochemical properties (oxidation and reduction potentials) and enabling the detection (recognition) of the analytes. For this reason, we recently focused on D–π–A-type pyridine–boron trifluoride (BF_3_) complexes as colorimetric and fluorescent sensors for water.^11^ In our previous work, we have designed and actually developed a D–(π–A)_2_-type pyridine–BF_3_ complex YNI-2-BF_3_ composed of a carbazole skeleton as a donor moiety and two pyridine–BF_3_ units as acceptor moieties (Fig. 1a).^11*a*^ It was found that the blue-shift of the photoabsorption and the enhancement of the fluorescence intensity in the low-water-content region could be attributed to the change in the ICT characteristics due to the dissociation of YNI-2-BF_3_ into the D–(π–A)_2_-type pyridine dye YNI-2 by water molecules. Furthermore, a red-shift of fluorescence bands with a decrease in the fluorescence intensity in the high-water-content region was observed because of the formation of the hydrogen-bonded proton transfer (PTC) complex YNI-2-H_2_O with water molecules. Moreover, 9-methyl pyrido[3,4-*b*]indole-BF_3_ complex, 9-MP-BF_3_, was designed and developed as a colorimetric and ratiometric fluorescent sensor for the detection of water in the low-, moderate-, and high-water-content regions in solvents (Fig. 1b).^11*b*^ It was found that in the low-water-content region, the blue-shifts of photoabsorption bands with an isosbestic point and fluorescence bands with an isoemissive point could be attributed to the dissociation of 9-MP-BF_3_ into 9-methyl pyrido[3,4-*b*]indole (9-MP) by water molecules. In the moderate-water-content region, the photoabsorption and the fluorescence bands of 9-MP gradually shifted to a longer wavelength region with the increase in the fluorescence intensity, which can be ascribed to the formation of the hydrogen-bonded complex (9-MP-H_2_O) with water molecules. Furthermore, in the high-water-content region, two photoabsorption bands and one fluorescence band gradually reappeared in a longer wavelength region with simultaneous decreases in the photoabsorption and the fluorescence bands of 9-MP-H_2_O, which was attributed to the formation of the PTC complex (9-MP-H^+^) with water molecules. Consequently, our previous works proposed that the ICT-type pyridine–BF_3_ complexes can act as colorimetric and fluorescent sensors for the detection of water in the low-, moderate-, and high-water-content regions in solvents.

In this work, in order to gain a further insight into the impacts of fluorophore and molecular structure on the optical sensing properties of ICT-type pyridine–BF_3_ complexes for the detection of water, we designed and developed propeller-structured 3,5,8-trithienyl-BODIPY ST-3 (ref. 15) and its pyridine–BF_3_ complex ST-3-BF_3_, which have three units of 2-(pyridin-4-yl)-3-(thiophen-2-yl)acrylonitrile as strong electron-withdrawing moiety at the 3-, 5-, and 8-positions on the BODIPY skeleton, leading to the bathochromic shift of the photoabsorption band due to the enhancement of the ICT characteristics (Fig. 1c). 4,4-Difluoro-4-bora-3*a*,4*a*-diaza-*s*-indacenes (boron dipyrromethene: BODIPY) dyes have created considerable interest as optical sensors and probes,^16^ photosensitizers^17^ for photodynamic therapy (PDT), and emitters^18^ and dye-sensitizers^19^ for optoelectronic devices such as organic light-emitting diodes (OLEDs) and dye-sensitized solar cells (DSSCs). It is expected that the addition of a trace amount of water to the solution of ST-3-BF_3_ causes the dissociation of ST-3-BF_3_ into ST-3 by water molecules, resulting in the photoabsorption and fluorescence spectral changes. Herein we report the preparation, the characterization, and the optical sensing properties of the propeller-structured 3,5,8-trithienyl-BODIPY-type pyridine–BF_3_ complex for the detection of a trace amount of water in acetonitrile based on FTIR, ^1^H and ^11^B NMR measurements, high-resolution mass spectrometry (HRMS) analysis, thermogravimetry-differential thermal analysis (TG-DTA), photoabsorption and fluorescence spectral measurements of ST-3-BF_3_ in acetonitrile containing various concentrations of water, and density functional theory (DFT) calculations.

## Results and discussion

### Characterization of ST-3-BF_3_

**Fig. 2:**
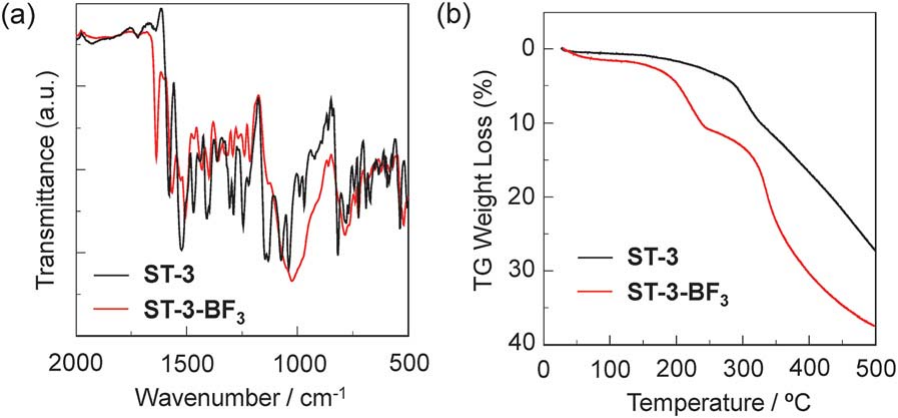
(a) FTIR spectra of ST-3 and ST-3-BF_3_. (b) TG curves for ST-3 and ST-3-BF_3_ at a heating rate of 10 °C min^−1^.

**Fig. 3:**
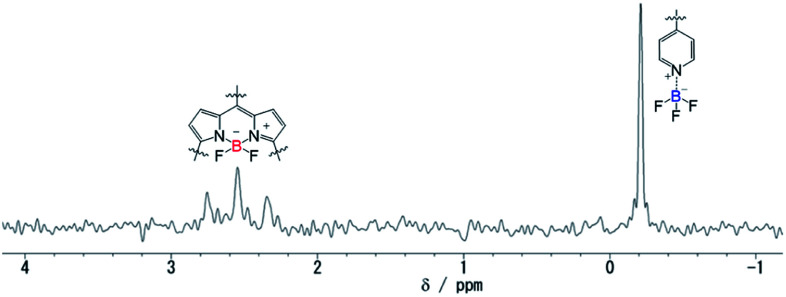
^11^B NMR spectrum of ST-3-BF_3_ in acetonitrile-*d*_3_.

**Fig. 4:**
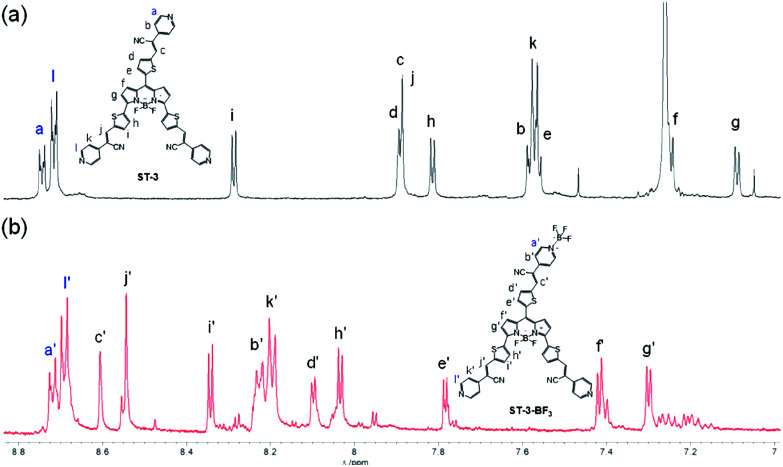
^1^H NMR spectra of (a) ST-3 in CDCl_3_ and (b) ST-3-BF_3_ in acetonitrile-*d*_3_.

The propeller-structured 3,5,8-trithienyl-BODIPY-type pyridine–BF_3_ complex ST-3-BF_3_ studied in this work was prepared by treating ST-3 (ref. 15) with boron trifluoride diethyl etherate (BF_3_–OEt_2_) and fully characterized by FTIR, ^1^H and ^11^B NMR measurements, HRMS, and TG-DTA, although we could not obtain the ^13^C NMR spectrum that is clear enough to be assigned, due to the low solubility of ST-3-BF_3_ into solvent (Fig. 2–4). In the FTIR spectra, the B–F and B–N stretching bands originating from the BODIPY core were observed at 1082 and 1522 cm^−1^ for ST-3 and 1047 and 1504 cm^−1^ for ST-3-BF_3_, respectively (Fig. 2a). In addition, for ST-3-BF_3_, the characteristic C=N stretching band of the pyridyl group coordinated to BF_3_, the B–N stretching band of the pyridine–BF_3_ complex, and the B–F stretching band of BF_3_ were clearly observed at 1636, 1429, and 1024 cm^−1^, respectively. The TG-DTA of ST-3-BF_3_ indicated the decreased weight loss by 7.78% in comparison with that of ST-3 at around 275 °C, which is in good agreement with the calculated weight loss of 7.61% for the release of a BF_3_ unit from ST-3-BF_3_ (Fig. 2b). Moreover, the ^11^B NMR spectrum of ST-3-BF_3_ in acetonitrile-*d*_3_ showed a singlet at −0.21 ppm, which can be assigned to BF_3_ coordinated to the pyridyl group, and a characteristic triplet with coupling constant (*J*_B–F_) of 33 Hz at around 2–3 ppm, which indicates the presence of the BF_2_ group in BODIPY (Fig. 3). Based on this result, the ratio of the peak integrals of BF_3_ and BF_2_ was 1 : 1. Obviously, the FTIR, TG-DTA, and ^11^B NMR results demonstrated the presence of one BF_3_ unit coordinated to the pyridyl group in ST-3-BF_3_, although HRMS (ESI) of ST-3-BF_3_ showed the base peak corresponding to the molecular ion for *m*/*z* of [ST-3 + 2H]^2+^ (calcd for C_45_H_27_N_8_BF_2_S_3_, 412.07855; found 412.07918) due to the measurement condition.

For the ^1^H NMR spectrum of the propeller-structured 3,5,8-trithienyl-BODIPY-type pyridine–BF_3_ complex, if it is assumed that BF_3_ coordinates to a pyridyl group at the end of the 3- or 5-position on the BODIPY core, the ^1^H NMR spectrum of ST-3-BF_3_ is expected to be more complex than that of ST-3. For example, the 1-position protons on the pyridyl groups at the end of the 3-, 5-, and 8-positions on the BODIPY core will appear as three different signals. On the other hand, if it is assumed that BF_3_ coordinates to the pyridyl group at the end of the 8-position on the BODIPY core, the signal pattern in the ^1^H NMR spectrum of ST-3-BF_3_ is expected to be similar to that of ST-3. In fact, the ^1^H NMR spectrum of ST-3-BF_3_ demonstrated that the chemical shifts and signal pattern of the 1-position protons (*H*_a′_ and *H*_l′_) on the pyridyl groups of ST-3-BF_3_ show little change from those (*H*_a_ and *H*_l_) of ST-3, indicating the formation of the pyridine–BF_3_ complex coordinated to the pyridyl group at the end of the 8-position on the BODIPY core (Fig. 4), although the comparison of the ^1^H NMR spectra between ST-3 and ST-3-BF_3_ might be difficult because different deuterated solvents were used for ST-3 (in CDCl_3_) and ST-3-BF_3_ (in acetonitrile-*d*_3_).

**Fig. 5:**
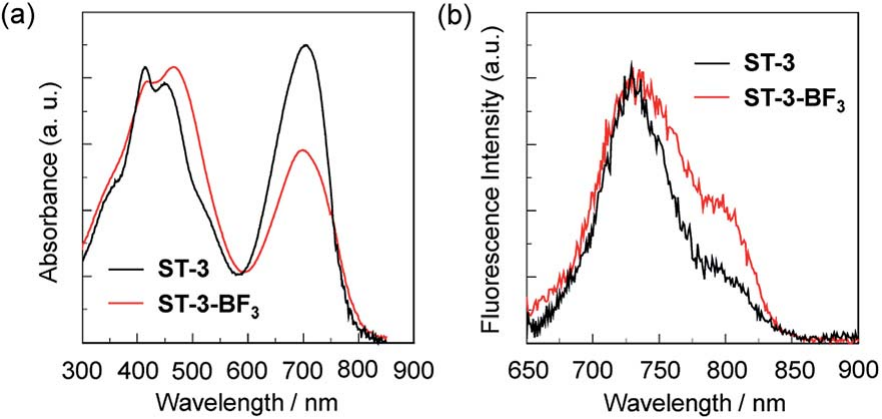
(a) Photoabsorption and (b) fluorescence (*λ*_ex_ = 640 nm) spectra of ST-3 and ST-3-BF_3_ in acetonitrile.

**Fig. 6:**
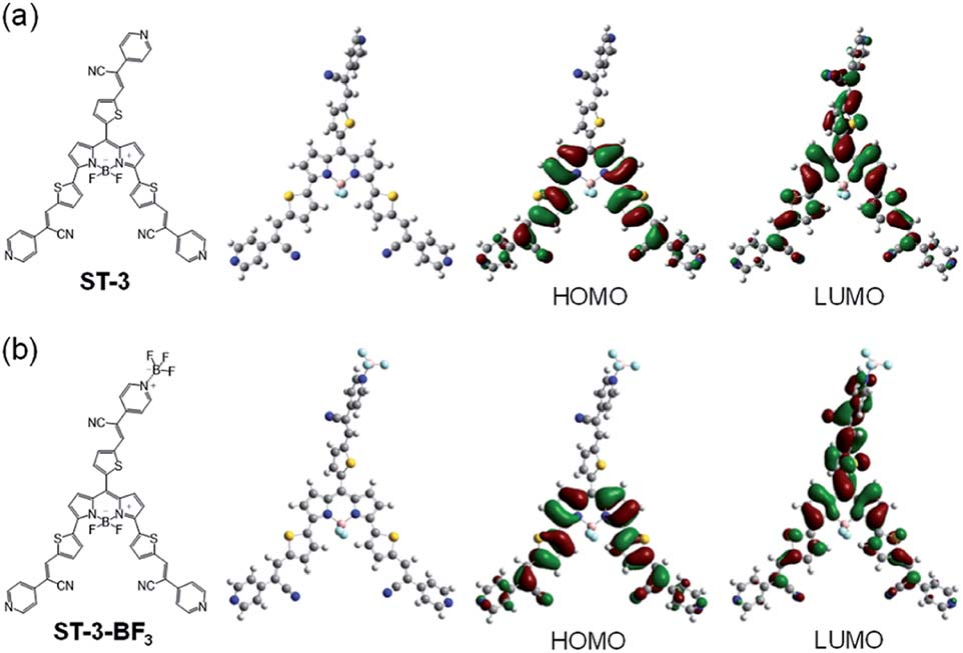
Optimized geometries, HOMOs, and LUMOs of (a) ST-3 and (b) ST-3-BF_3_ derived from DFT calculations at the B3LYP/6-31G(d,p) level.

The photoabsorption spectra of ST-3 and ST-3-BF_3_ in acetonitrile revealed that the two dyes show a strong and broad photoabsorption band in the range of 600 to 800 nm, which is assigned to the S_0_ → S_1_ transition of the BODIPY skeleton with the expanded π-conjugated system over the 2-(pyridin-4-yl)-3-(thiophen-2-yl)acrylonitrile units at the 3-, 5-, and 8-positions onto the BODIPY core (Fig. 5a). In addition, a photoabsorption band was also observed in the range of 300 to 550 nm, which can be assigned to the ICT band between the 2-(pyridin-4-yl)-3-(thiophen-2-yl)acrylonitrile units at 3-, 5-, and 8-positions and the BODIPY core.^15,20^ It is worth noting here that for ST-3, the peak absorbance of the former photoabsorption band at 695 nm is comparable with that of the latter ICT band at 415 nm, while for ST-3-BF_3_, the peak absorbance of the former band at 695 nm is lower than that of the latter band at 415 nm, which is attributed to the enhanced ICT characteristics. Moreover, for ST-3, the peak absorbance at 415 nm is higher than that at 450 nm, whereas for ST-3-BF_3_, the peak absorbance at 415 nm is lower than that at 465 nm. The corresponding fluorescence spectra of the two dyes show a characteristic fluorescence band at around 730 nm originating from the BODIPY skeleton, and the fluorescence band of ST-3-BF_3_ is broader than that of ST-3 (Fig. 5b). Consequently, the characterization of the propeller-structured 3,5,8-trithienyl-BODIPY-type pyridine–BF_3_ complex is successfully determined by the photoabsorption and fluorescence spectral measurements as well as FTIR, ^1^H and ^11^B NMR, HRMS, and TG-DTA. In order to examine the electronic structures of the propeller-structured 3,5,8-trithienyl-BODIPY dyes, the molecular structures and molecular orbitals of ST-3 and ST-3-BF_3_ were calculated using DFT at the B3LYP/6-31G(d,p) level (Fig. 6). For the two dyes, the HOMOs are mostly localized on the BODIPY core and the two thienyl groups at the 3- and 5-positions. On the other hand, the LUMO of ST-3 is mostly localized on the BODIPY core and the three thienyl groups at the 3-, 5-, and 8-positions, but that of ST-3-BF_3_ is mostly localized not only on the BODIPY core and the two thienyl groups at the 3- and 5-positions but also over the 2-(pyridin-4-yl)-3-(thiophen-2-yl)acrylonitrile unit at the 8-position. Thus, the DFT calculations suggest that the dissociation of ST-3-BF_3_ into ST-3 by water molecules results in the photoabsorption and fluorescence spectral changes based on their ICT characteristics due to the perturbation in the LUMO over the 2-(pyridin-4-yl)-3-(thiophen-2-yl)acrylonitrile unit of ST-3-BF_3_.

### Optical sensing ability of ST-3-BF_3_ for water in acetonitrile

**Fig. 7:**
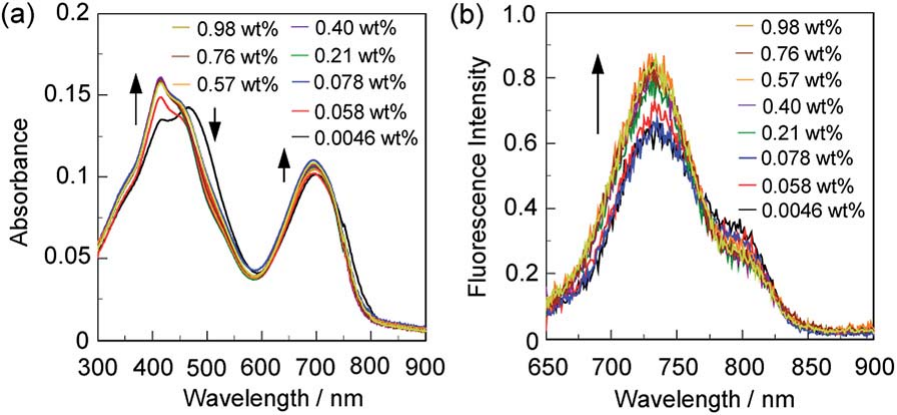
(a) Photoabsorption and (b) fluorescence spectra (*λ*_ex_ = 640 nm) of ST-3-BF_3_ (*c* = 2.5 × 10^−6^ M) in acetonitrile containing water (0.0046–0.98 wt%).

**Fig. 8:**
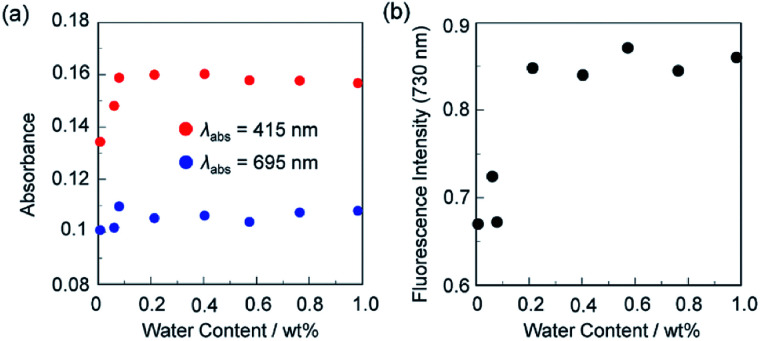
(a) Absorbance at 415 and 695 nm, and (b) fluorescence peak intensity at around 730 nm (*λ*_ex_ = 640 nm) of ST-3-BF_3_ as a function of water content below 1.0 wt% in acetonitrile.

In order to investigate the optical sensing ability of ST-3-BF_3_ for water in acetonitrile, the photoabsorption and fluorescence spectra of ST-3-BF_3_ were measured in acetonitrile that contained various concentrations of water (Fig. 7). With the increase in the water content in acetonitrile solution, a red-shift of the photoabsorption band at 465 nm with a decrease in the absorbance and simultaneous increases in the absorbance of the two photoabsorption bands at around 415 and 695 nm were observed, which could be ascribed to the dissociation of ST-3-BF_3_ into ST-3 by water molecules (Fig. 7a). On the other hand, the corresponding fluorescence spectra of ST-3-BF_3_ underwent an increase in the intensity of the fluorescence band at around 730 nm (Fig. 7b). To estimate the sensitivity and accuracy characteristics of ST-3-BF_3_ for the detection of water in acetonitrile, the changes in the absorbance and fluorescence intensity were plotted against the water fraction in acetonitrile (Fig. 8). The plots of absorbance in the water content region below 1.0 wt% demonstrated that the absorbance at around 415 nm increased linearly as a function of the water content, but the absorbance at around 695 nm slightly increased as a function of the water content (Fig. 8a). Moreover, the plot of fluorescence intensity at around 730 nm in the water content region below 1.0 wt% demonstrates that the fluorescence peak intensity increases almost linearly as a function of the water content (Fig. 8b). The increases in the absorbance and fluorescence intensity leveled off in the water content region above 0.2 wt%. Thus, it was found that the addition of a trace amount of water to the acetonitrile solution of ST-3-BF_3_ causes the change in the ICT characteristics due to the dissociation of ST-3-BF_3_ into ST-3 by water molecules, and as the result, the photoabsorption band at around 415 nm and the fluorescence band at around 730 nm increase linearly as a function of the water content below only 0.2 wt%. Consequently, this work demonstrated that the 3,5,8-trithienyl-BODIPY-type pyridine–BF_3_ complex can act as a high-sensitive optical sensor for the detection of a trace amount of water in acetonitrile.

## Conclusions

We have designed and developed the propeller-structured 3,5,8-trithienyl-BODIPY-type pyridine–boron trifluoride complex, ST-3-BF_3_, which has three units of 2-(pyridin-4-yl)-3-(thiophen-2-yl)acrylonitrile at the 3-, 5-, and 8-positions on the BODIPY skeleton, as an intramolecular charge transfer (ICT)-type optical sensor for the detection of a trace amount of water in acetonitrile. It was found that the addition of a trace amount of water to the acetonitrile solution of ST-3-BF_3_ causes the photoabsorption and fluorescence spectral changes based on the ICT characteristics due to the dissociation of ST-3-BF_3_ into ST-3 by water molecules. Indeed, the absorbance and fluorescence intensity increased linearly as a function of the water content below only 0.2 wt%. Based on the optical sensing mechanism of ST-3-BF_3_, we demonstrated that the 3,5,8-trithienyl-BODIPY-type pyridine–boron trifluoride complex can act as a high-sensitive optical sensor for the detection of a trace amount of water in acetonitrile. Thus, our continuous works regarding optical sensors for water confirm that the ICT-type pyridine–boron trifluoride complex is one of the most promising colorimetric and fluorescent sensors for the detection of water in the low-, moderate-, and high-water-content regions in solvents. Moreover, NIR dyes such as ICT-type pyridine–boron trifluoride complex which make it possible to control the intensity of NIR luminescence by the presence or absence of water, may be applicable to the wavelength conversion dye-doped films for controlling the plant growth (photomorphogenesis).

## Experimental

### General

IR spectra were recorded on a SHIMADZU IRTracer-100 using ATR method. ^1^H NMR and ^11^B NMR spectra were recorded on a Varian-500 (500 MHz) FT NMR spectrometer. High-resolution mass spectral data by ESI were acquired on a Thermo Fisher Scientific LTQ Orbitrap XL. Photoabsorption spectra were observed with a SHIMADZU UV-3150 spectrophotometer. Fluorescence spectra were measured with a Hitachi F-4500 spectrophotometer. Super dehydrated acetonitrile was used for all the experiments. The addition of water to acetonitrile solutions containing ST-3-BF_3_ was made by weight percent (wt%). The determination of water in acetonitrile was done with an MKC-610 and MKA-610 Karl Fischer moisture titrator (Kyoto Electronics manufacturing Co., Ltd.) based on Karl Fischer coulometric titration.

### Synthesis

#### (2Z,2′Z,2′′Z)-3,3′,3′′-((5,5-difluoro-5*H*-4l4,5l4-dipyrrolo[1,2-*c*:2′,1′-*f*][1,3,2]diazaborinine-3,7,10-triyl)tris(thiophene-5,2-diyl))tris(2-(pyridin-4-yl)acrylonitrile)-boron trifluoride complex (ST-3-BF_3_)

To a solution of ST-3 (ref. 15) (5.0 mg, 6.1 μmol) in acetonitrile (5.0 mL) under a nitrogen atmosphere was added dropwise 47% BF_3_–OEt_2_ (4.6 μL, 37 μmol) diluted with acetonitrile (1.0 mL) for 10 min, and then, the solution was stirred for 3 h at room temperature. Next, to toluene was added dropwise the reaction mixture, and then, the resulting precipitate was filtered to give ST-3-BF_3_ (4.0 mg, 74% yield) as a black solid; FT-IR (ATR): *ν̃* = 1636 (C=N str. for pyridyl group coordinated to BF_3_), 1504 (B–N str. for BODIPY core), 1429 (B–N str. for pyridine–BF_3_ complex), 1047 (B–F str. for BF_2_ in BODIPY core), 1024 (B–F str. for BF_3_) cm^−1^; ^1^H NMR (500 MHz, acetonitrile-*d*_3_): *δ* = 7.30 (d, *J* = 4.5 Hz, 2H), 7.42 (d, *J* = 4.7 Hz, 2H), 7.78 (d, *J* = 4.0 Hz, 1H), 8.03 (d, *J* = 4.3 Hz, 2H), 8.10 (d, *J* = 3.8 Hz, 1H), 8.20 (d, *J* = 7.0 Hz, 4H), 8.23 (d, *J* = 7.5 Hz, 2H), 8.34 (d, *J* = 4.3 Hz, 2H), 8.54 (s, 2H), 8.61 (s, 1H), 8.69 (d, *J* = 7.0 Hz, 4H), 8.72 (d, *J* = 7.1 Hz, 2H) ppm; ^11^B NMR (160 MHz, acetonitrile-*d*_3_) *δ* = −0.21 (s), 2.55 (t, *J*_B–F_ = 33 Hz) ppm; HRMS (ESI): *m*/*z* (%): [M + 2H]^2+^ calcd for C_45_H_27_N_8_BF_2_S_3_, 412.07855; found 412.07918.

## Conflicts of interest

There are no conflicts to declare.

## Supplementary Material

RA-010-D0RA06569B-s001
