# Supplementary material for: Development of optical sensor for water in acetonitrile based on propeller-structured BODIPY-type pyridine–boron trifluoride complex
Source: RSC Adv. 2020 Sep 14;10(56):33836–43. doi: 10.1039/d0ra06569b (PMC9056773; doi:10.1039/d0ra06569b)
Supplement: RA-010-D0RA06569B-s001 [file RA-010-D0RA06569B-s001.pdf]

Electronic Supplementary Material (ESI) for RSC Advances.
This journal is © The Royal Society of Chemistry 2020

Supplementary Information

Development of optical sensor for water in acetonitrile based on propeller-structured BODIPY-type pyridine-boron trifluoride complex

Shuhei Tsumura, Kazuki Ohira, Keiichi Imato* and Yousuke Ooyama*

Department of Applied Chemistry, Graduate School of Engineering, Hiroshima University, Higashi-Hiroshima, 739-8527, Japan.

Fax: +81 82 424 5494; Tel: +81 82 424 7689; E-mail: kimato@hiroshima-u.ac.jp; yooyama@hiroshima-u.ac.jp

(a)

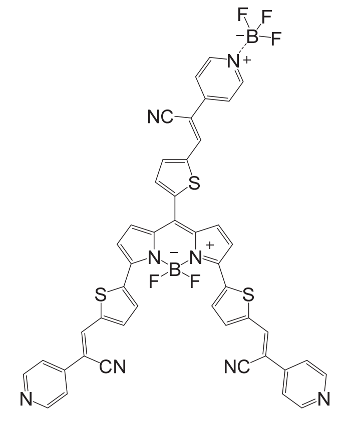

ST-3-BF$_3$

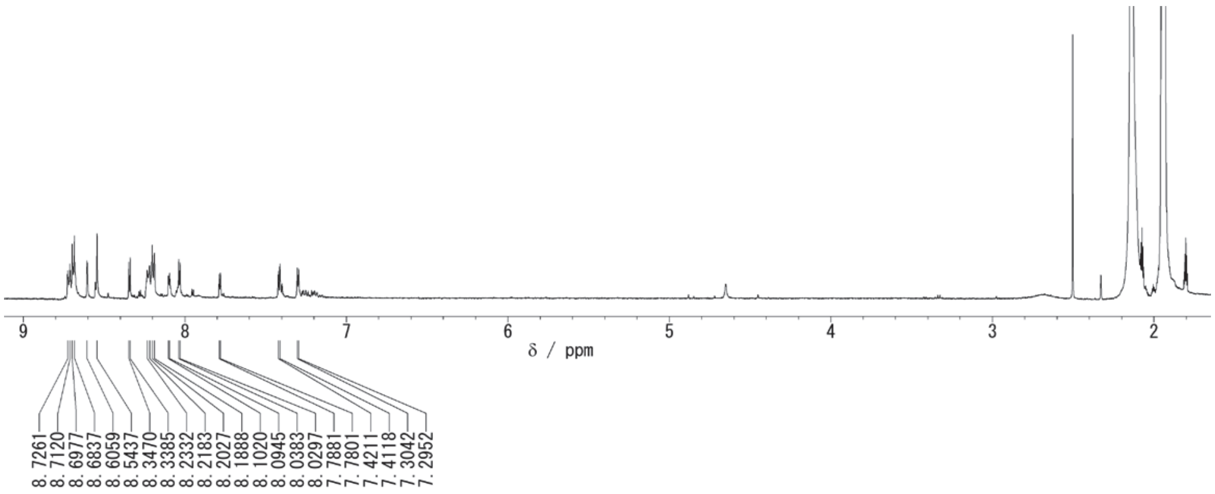

(b)

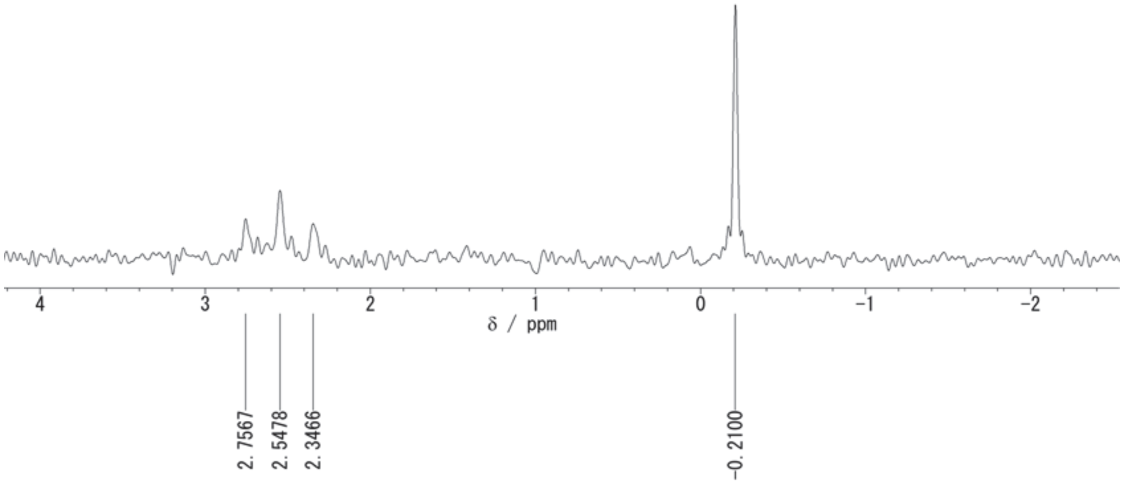

Fig. S1 (a) ^{1}H NMR (500 MHz) and (b) ^{11}B NMR (160 MHz) spectra of **ST-3-BF$_3$** in acetonitrile-d_3.
